# New Insights Into Novel Therapeutic Targets in ANCA-Associated Vasculitis

**DOI:** 10.3389/fimmu.2021.631055

**Published:** 2021-04-01

**Authors:** Yuji Nozaki

**Affiliations:** Department of Hematology and Rheumatology, Kindai University Faculty of Medicine, Osaka-sayama, Japan

**Keywords:** anti-neutrophil cytoplasmic autoantibody, anti-neutrophil cytoplasmic autoantibody-associated vasculitis, biologics, cytokine, cytokine-immunological terms

## Abstract

Biologics targeting inflammation-related molecules in the immune system have been developed to treat rheumatoid arthritis (RA), and these RA treatments have provided revolutionary advances. Biologics may also be an effective treatment for anti-neutrophil cytoplasmic autoantibody (ANCA)-associated vasculitis, particularly in patients with resistance to standard treatments. Despite the accumulation of clinical experience and the increasing understanding of the pathogenesis of vasculitis, it is becoming more difficult to cure vasculitis. The treatment of vasculitis with biologics has been examined in clinical trials, and this has also enhanced our understanding of the pathogenesis of vasculitis. A humanized anti-interleukin-5 monoclonal antibody known as mepolizumab was recently demonstrated to provide clinical benefit in the management of eosinophilic granulomatosis with polyangiitis in refractory and relapsing disease, and additional new drugs for vasculitis are being tested in clinical trials, while others are in abeyance. This review presents the new findings regarding biologics in addition to the conventional immunosuppressive therapy for ANCA-associated vasculitis.

## Introduction

Anti-neutrophil cytoplasmic autoantibodies (ANCAs) are the major serological markers of primary systemic necrotizing small vessel inflammation or ANCA-associated vasculitis (AAV), including granulomatosis with polyangiitis (GPA), microscopic polyangiitis (MPA), and eosinophilic granulomatosis with polyangiitis (EGPA) (1, 2). ANCAs for proteinase 3 (PR3) are more prevalent in patients with GPA, and ANCAs for myeloperoxidase (MPO) are more prevalent in patients with MPA, but there is substantial overlap. Regarding the clinical utility of ANCAs’ specificity in the classification of the forms of AAV, ANCA specificity is more likely to be associated with the patient’s genetic predisposition (3), treatment effect(s) (4), the risk of recurrence (5, 6), and the prognosis (7) than the clinical diagnosis. Distinct cytokine profiles were identified for PR3-AAV and MPO-AAV with GPA, MPA, and EGPA (8).

These differences in circulating immuno-mediators are strongly associated with ANCA specificity, not clinical diagnosis, and the heterogeneity of AAV subtypes is associated with the clinical phenotypes identified in the traditional clinical classification of GPA and MPA (8). Clinical trial results and clinical practice data have formed the foundation of the management of AAV, which is based on the disease severity. In 2016, the European League Against Rheumatism (EULAR) updated their recommendations for the management of primary small- and medium-vessel vasculitis, including the management of AAV (9). Glucocorticoids are a central component of the management of AAV in induction and maintenance therapy and are not sufficient by themselves, especially in the context of organ invasion. For active AAV, the current treatment recommendation is to first administer high doses of glucocorticoids, followed by a gradual decrease in steroids (10).

Cyclophosphamide is also used in combination with steroids to induce remission in AAV, but its metabolites are toxic to the bladder and reproductive organs and may cause malignancy and infertility in the long term (11). In non-immediately life-threatening AAV, there was no significant difference in remission rates between treatment groups receiving daily oral versus intravenous pulsed cyclophosphamide therapy regimens, and the total dose of cyclophosphamide was reduced in the intravenous pulse group (11).

Advances in induction therapy have transformed AAV from a life-threatening disease to a chronic disease with relapse. Relapse is not uncommon, occurring in 30%–50% of patients with AAV within 5 years of onset, and often 12–18 months after treatment with immunosuppressive agents is discontinued (12). In AAV, maintenance therapy is recommended to prevent relapse after achieving remission by the induction therapy. Maintenance immunosuppressive agents such as cyclophosphamide, mycophenolate mofetil, and azathioprine are used in combination to prevent relapse after the successful induction of remission. More recently, biologic agents have begun to play an important role in the induction of clinical remission and the maintenance of remission in severe AAV. The biologic rituximab is indicated for remission induction and the management of severe and relapsed GPA and MPA, and data suggest a role for pre-emptive fixed-interval rituximab maintenance therapy in remission treatment (13, 14). Treatment with the biologic mepolizumab also provided a significantly greater number of weeks of remission and higher remission rates than a placebo when it was used as maintenance therapy for EGPA (15). Other biologics are either being tested in clinical trials or have failed because their effectiveness could not be verified, or they produced unacceptable side effects. However, it is possible that using biologics could reduce the rate of side effects caused by steroids in the treatment of AAV, by providing a new mechanism of action. This review presents new insights into novel therapeutic targets in AAV.

## Review

### ANCAs

ANCAs are autoantibodies against cytoplasmic antigens expressed on the primary granules of neutrophils and the lysosomes of monocytes. The primary granules of neutrophils contain a series of antimicrobial proteins, including lysozyme, MPO, neutral serine proteinases (PR3, elastase, and cathepsin G), and acid hydrolases (cathepsins B and D). Autoantibodies can develop against any of these proteins, but the most clinically important antibodies are against MPO and PR3. During the active phase of AAV, ANCA is usually immunoglobulin G (IgG), but other immunoglobulin classes (IgM and IgA) have also been reported. PR3-ANCA is most frequently associated with GPA (75%), and MPO-ANCA is associated with MPA (60%) and EGPA (30%). MPO-ANCA is also associated with renal limited vasculitis (80%) (**Table 1**) (16, 17).

**Table 1:** The positive rate of ANCA in vasculitis.

ANCA-associated vasculitis	MPO-ANCA	PR3-ANCA
GPA	20%	75%
MPA	60%	30%
EGPA	30%	5%
Renal limited vasculitis	80%	10%
Drug-induced vasculitis	90%	10%

ANCA, antineutrophil cytoplasmic autoantibodies; EGPA, eosinophilic granulomatosis with polyangiitis; GPA, granulomatosis with polyangiitis; MPA, microscopic polyangiitis; MPO, myeloperoxidase; PR3, proteinase 3.

Atypical ANCAs, which do not react with either PR3 or MPO (positive by indirect immunofluorescence and negative by enzyme-linked immunosorbent assay), have been identified in a range of nonvasculitic conditions: inflammatory bowel diseases, autoimmune diseases, and malignancies.

PR3- and MPO-ANCA have also been found in chronic infections such as endocarditis, tuberculosis, human immunodeficiency virus, hepatitis C, and bartonellosis. The presence of both anti-MPO and anti-PR3 antibodies in the same patient is very rare and suggests drug-induced vasculitis (16). In a subgroup of patients (10%) whose clinical and pathological features are consistent with AAV, the test result for ANCA remains negative. Although these patients may have a similar clinical course and response to treatment, ANCA-negative patients are more likely to have renal-limited disease or less severe systemic disease (16).

### Cytokine Production

#### Th1/Th2

**Figure 1:**
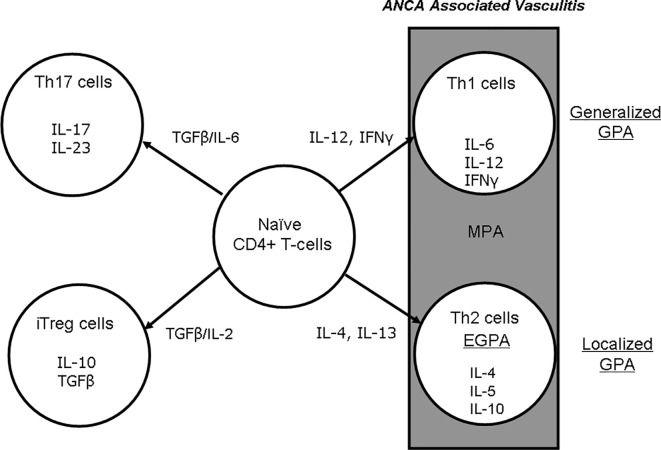
Differentiation of naïve CD4+ T cells to Th1, -2, -17, and iTreg cells and effects on cytokine production in AAV. AAV, ANCA-associated vasculitis; ANCA, antineutrophil cytoplasmic antibody; EGPA, eosinophilic granulomatosis with polyangiitis; GPA, granulomatosis with polyangiitis; IFNγ, interferon gamma; IL, interleukin; iTreg, inducible regulatory T cell; MPA, microscopic polyangiitis; TGFβ, transforming growth factor beta.

Berti et al. reported that the circulating cytokine profiles were significantly different between patients with PR3-ANCA and those with MPO-ANCA (8), and they noted that compared to the PR3-ANCA group, nine biomarkers were higher in their MPO-AAV group: interleukin (IL)-6, -15, and -18, granulocyte-macrophage colony-stimulating factor, chemokine (C-X-C) ligand 8/IL-8, chemokine (C-C motif) ligand 17/THYMUS, activation-regulated chemokine, and IL-18 binding protein. In contrast, four biomarkers were higher in MPO-AAV than in PR3-AAV: soluble IL-6 receptor, soluble tumor necrosis factor (TNF) receptor II, neutrophil gelatinase-associated lipocalin, and soluble intercellular adhesion molecule-1. In active AAV, the cellular infiltration in kidney, lung, and nasal tissues is composed mainly of macrophages, T cells, and B cells (18). In a validation study of T-cell markers in 38 renal biopsies from patients with AAV, Kidder et al. observed an increased number of CD8+ T cells in the periglomerular and interstitial areas in kidneys with a crescent-shaped histology. They also reported a significant correlation between the number of CD8+ T cells and the glomerular filtration rate. Despite the low number of T lymphocytes infiltrating the glomerulus, CD8+ T cells were more predominant than CD4+ T cells (19). In contrast, T cells stimulated with MPO showed much less or no proliferative response in both patients and healthy controls (20). Thus, at least in GPA, the pathogenic role of T cells at the effector stage of AAV is not fully established, because CD4+ T cells, based on their cytokine profile and associated functions, have been shown to have two distinct types: Th1 and Th2 cells (**Figure 1**). In localized GPA, T cells in nasal inflammatory infiltrates were shown to express the Th1 marker CD26 (21). In addition, more interferon-alpha (IFNα)-positive cells were detected in the nasal inflammatory infiltrate in localized GPA than in generalized GPA. The tissues of eosinophilic infiltration and eosinophilia are also common in GPA and EGPA, and thus polarization to the Th2 profile would be expected in EGPA.

Those findings were accompanied by increases in spontaneous IFNα and IL-10 produced by peripheral blood mononuclear cells in patients with localized GPA compared to generalized GPA, whereas high levels of IL-4 mRNA were detected in the nasal inflammatory infiltrates of the patients with generalized GPA. A Th2 environment in this phenomenon was confirmed in nasal granulomas of systemic GPA. In immunohistochemistry staining, IFNα was not detected in nasal biopsies of 10 patients with systemic active GPA, but IL-4 was upregulated (22). These cells are likely to be T cells, especially Th2 cells and eosinophils. These data thus support potential differences in Th responses between local and systemic GPA in nasal granulomatous lesions. Csernok et al. reported the expression of IFNα mRNA in nasal granulomas with systemic GPA, but IL-4 mRNA was only expressed in two of five patients (23). Komocsi et al. also demonstrated Th1-like cytokine production and features suggestive of CD4+ T-cell-mediated cytotoxicity. In GPA, CD4+CD28- T cells are recruited from the blood to granulomatous lesions *via* interaction with CD18, followed by cytokine secretion to promote monocyte accumulation and granuloma formation (24).

#### Th17

The Th17 T helper subset is involved in the defense against extracellular bacteria and fungi and has been implicated in autoimmune diseases (25). Th17 cells express the transcription factor RORγt (retinoic acid-related orphan receptor gamma t) and produce IL-17A–F, which are centered on IL-17A (26). IL-23 is responsible for the expansion and maturation of the Th17 subset (27). Th17 cells are also inhibited by Th1 and Th2 cytokines.

IL-17A acts on monocytes/macrophages and functions directly *via* ligation to the receptors expressed on monocytes (28). In peripheral blood, IL-17A induces the release of pro-inflammatory mediators from macrophages, and IL-17A induces a high percentage of Th17 cells in patients with AAV compared to healthy controls (29). Thus, IL-17A, as an important regulator and initiator of inflammation, mediates both innate and adaptive immunity. IL-17A not only defines the Th17 subset; it is also a logical therapeutic target for diseases induced by Th17-dominant autoimmune responses.

Nogueira et al. described that in a cohort of 28 patients with acute AAV and 65 patients in AAV, serum IL-17A levels were significantly higher than in healthy controls, with a similar pattern for serum IL-23 levels (30). Other researchers have found no evidence of this expansion Th17 response in AAV (31, 32). For example, Krohn et al. detected no difference in serum IL-17A levels between 70 AAV patients and the healthy controls, but interestingly, they observed a significant increase in IL-17C levels (33). Velden et al. demonstrated the presence of IL-17 in kidney tissues by immunohistochemical staining, and they reported that the percentage of Th1-like Th17 cells was higher in patients in the acute phase of the disease or in untreated remission compared to healthy controls (34). Thus, IL-17A, as an important regulator and initiator of inflammation, mediates both innate and adaptive immunity. Neutrophils can also induce Th17 cell chemotaxis, making this cell axis even more interesting as a potential target in the treatment of AAV (35).

#### C5 and C5a Receptor

C5a, an anaphylatoxin of complement, is a potent inflammatory mediator (36). Alternative classical and lectin pathways converge on the activation of C5, releasing C5a and C5b. C5a is a potent chemoattractant for neutrophils, and ligation of C5aR/CD88 by C5a activates neutrophils. Neutrophil priming increases the availability of ANCA antigens at the surface, where they interact with ANCA and activate neutrophils. When stimulated with proinflammatory cytokines, the terminal C5a/C5aR axis is activated, generating an automatic amplification loop that triggers an acute necrotizing vasculitis process from the primed neutrophils (resulting from their interaction with ANCA) (37). C5a acting on C5aR is a potent neutrophil chemoattractant and agonist (38), increases neutrophil adhesion, induces neutrophil degranulation, and generates reactive oxygen intermediates; C5a activates vascular endothelial cells *via* the C5aR, promoting cell retraction and increasing vascular permeability (39). Historically, the role of complement was thought to be limited in AAV, as renal biopsies rarely showed complement deposition and the absence of hypocomplementemia. Other clinical studies have supported these findings by showing activation of the alternative pathway in the cardiovascular system and deposition of complement components of the alternative pathway in tissues. Active AAV patients with renal involvement had higher levels of C3a and C5a in the circulation (40). These results could be the potential target in the treatment of AAV.

### Treatment Strategy for AAV

#### Immunosuppressive Agents

##### Cyclophosphamide

Cyclophosphamide is associated with reduced ovarian reserve, ovarian failure, and male infertility (41–45). A review of the prednisolone reduction regimens published in major trials has shown that on average, the cyclophosphamide doses 10 mg (after 19 weeks) and 7.5 mg (after 21 weeks) have been achieved (11, 46–51). Cyclophosphamide is usually administered orally or as pulse therapy for 3–6 months, and after remission is achieved, the cyclophosphamide is changed to a less-toxic agent. Intravenous cyclophosphamide pulse therapy may allow the reduction of the cumulative dose and consequently the toxicity. The strategy was demonstrated in the CYCLOPS study (11). Remission rates in patients with systemic but not immediately life-threatening AAV were not significantly different between those who received daily oral cyclophosphamide and those who received pulsed cyclophosphamide. With the pulsed cyclophosphamide regimen, patients could receive a cumulative dose of cyclophosphamide half that of the daily oral cyclophosphamide regimen, but the dose of corticosteroids remained the same. However, pulsed cyclophosphamide is associated with a higher risk of recurrence than daily oral cyclophosphamide (52).

##### Methotrexate and Mycophenolate Mofetil

Methotrexate (20-25 mg/week, orally or parenterally) can be used in place of cyclophosphamide in patients with less severe disease and normal renal function (53–62). There have been trials using either methotrexate or mycophenolate mofetil as remission-inducing agents in patients with AAV (60–62). Therefore, methotrexate should only be considered in cases of non-organ-threatening disease. So far, the induction trials using methotrexate are generally larger and have a higher grade of evidence than trials using mycophenolate mofetil. The two randomized controlled trials (RCTs) with mycophenolate mofetil to date have been conducted primarily in patients with MPA (61, 62). MPA often affects renal function, and methotrexate is not indicated in such situations. These studies did not include patients with pulmonary hemorrhage or central nervous system involvement, and mycophenolate mofetil should not be used routinely in life-threatening situations.

### Biological Agents

**Figure 2:**
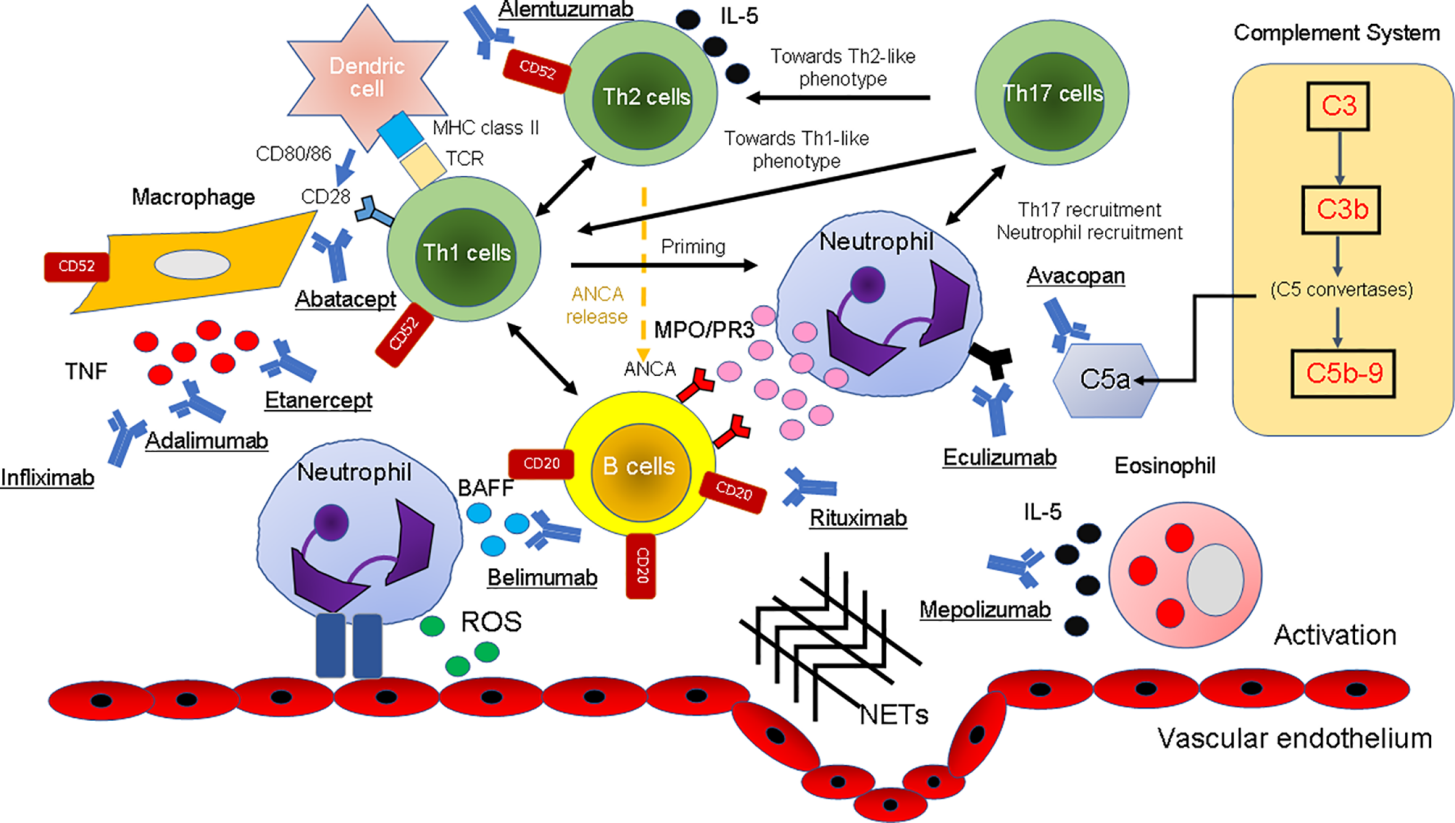
The pathogenesis of AAV and the inhibition of the binding of biological agents. The mechanism of the onset of AAV in the immune and complement systems in various cell types is illustrated. *T cells:* Etanercept, adalimumab, and infliximab as TNF-α inhibitors and alemtuzumab as an anti-CD52 inhibitor have been used for the treatment of AAV because they block the cytokine signaling pathway. By containing CTLA4, abatacept blocks the engagement of CD28 on the surface of T cells by B7.1 (CD80) or B7.2 (CD86) on the surface of APCs or B cells with its ligand, thereby inhibiting T-cell activation. *B cells:* B cells also act as APCs for T lymphocytes, and they produce pro-inflammatory cytokines that are useful for T-cell hyperactivity and neutrophil priming. Immunotherapies targeting B cells (depletion of B cells with rituximab and blockade of BLyS by belimumab) reduce the recruitment of these effector cells at the site of immune complex deposition, thereby reducing inflammation and tissue damage. *Eosinophils:* IL-5 is produced by Th2 cells and induces differentiation and maturation of human eosinophils. By neutralizing IL-5, mepolizumab inhibits the IL-5 signaling pathway and may be a therapeutic option for patients with EGPA. *Complement system:* Eculizumab and avacopan bind C5 and C5aR with picomolar affinity and inhibit the enzymatic activation by C5 convertases. *Others:* The binding of ANCA to these autoantigens activates neutrophils attached to the vascular endothelium. Degranulation of neutrophils releases ROS and NETs that damage the vascular endothelium. AAV, ANCA-associated vasculitis; ANCA, antineutrophil cytoplasmic antibody; APCs, antigen-presenting cells; BAFF, B-cell activating factor inhibitor; BLys, B-lymphocyte stimulator; NETs, neutrophil extracellular traps; ROS, reactive oxygen species; EGPA, eosinophilic granulomatosis with polyangiitis; IL, interleukin; MPO, myeloperoxidase; PR3, proteinase 3; TNF, tumor necrosis factor.

Representative clinical trials and the pathogenesis of AAV by the inhibition of binding of biological agents for AAV with biologics are shown in **Table 2** and **Figure 2**.

**Table 2:** Clinical trials with biological agents in ANCA associated vasculitis.

Drug(s)	Status	Allocation	n	Inclusion criteria	Primary endpoint	Trial no.	Last Update Posted	Public-ation results
Rituximab	Completed	RCT	197	GPA	Disease remission for 6 mos.	NCT00104299	4/21/2017	13
Rituximab	Completed	RCT	44	AAV with renal involvement	Disease remission and rates of relapse at 24 mos.	ISRCTN28528813	3/6/2015	65
Abatacept	Terminated	RCT	7	AAV	Relapse rate over 24 mos.	NCT00482066	3/29/2015	
Abatacept	Recruiting	RCT	63	GPA	Reduce the treatment failure rate for 12 mos.	NCT02108860	3/3/2019	
Abatacept	Completed	N/A	20	GPA	Adverse events up to 3 yrs + 4 mos.	NCT00468208	1/18/2016	86
Belimumab	Completed	RCT	106	GPA and MPA	Time to first relapse up to 4 yrs	NCT01663623	4/17/2018	75
Belimumab + Rituximab	Recruiting	RCT	30	AAV with PR3 ANCA positivity	Time to PR3 ANCA negativity	NCT03967925	6/9/2020	
Infliximab/ Rituximab	Completed	N/A	20	AAV	Partial or complete remission of the vasculitis	NCT00307593	11/19/2007	
Infliximab	Completed	Non- RCT	37	GPA, MPA, and renal limited vasculitis	Disease remission for 52 wks	NCT00753103	9/16/2008	
Alemtuzumab	Unknown	RCT	24	GPA	Response and a severe adverse event for 6 mos.	NCT01405807	7/29/2011	
Etanercept	Completed	RCT	180	GPA	Sustained remissions for 27mos.	NCT00005007	12/28/2007	79
Avacopan	Completed	RCT	300	AAV	Disease remission for 26 wks	NCT02994927	6/22/2020	97
Avacopan	Completed	RCT	42	AAV	Disease remission at 12 wks	NCT02222155	11/16/2016	95
Avacopan	Completed	RCT	67	AAV	Achieving ≥50% reduction in disease activity at 12 wks	NCT01363388	6/27/2020	96
Eculizumab	Withdrawn	RCT	0	AAV	Change in disease activity as measured at 12 wks	**NCT01275287**	2/23/2017	
Mepolizumab	Active, not recruiting	Case Only	300	EGPA	Adverse events up to 2 yrs	NCT03557060	9/10/2018	
Mepolizumab	Completed	RCT	136	EGPA	Each category of accrued duration of remission for 52 wks	NCT02020889	1/31/2018	15
Mepolizumab	Completed	N/A	10	EGPA	Attain remission rate for 52 wks	NCT00716651	6/15/2012	
Mepolizumab	Completed	N/A	10	EGPA	Adverse events for approx. 44 wks	NCT00527566	3/22/2017	
Benralizumab/ Mepolizumab	Recruiting	RCT	140	EGPA	Remission rate at 36 and 48 wks	NCT04157348	10/8/2020	

AAV, ANCA-associated vasculitis; EGPA, eosinophilic granulomatosis with polyangiitis; GPA, granulomatosis with polyangiitis; mos., months; MPA, microscopic polyangiitis; N/A, not available; PR3 ANCA, proteinase 3 antineutrophil cytoplasmic antibody; RCT, randomized controlled trial; wks, weeks; yrs, years.

#### Anti-CD20 Monoclonal Antibody

A rationale for B-cell activation in AAV has been based on the pathogenicity of ANCA (63). B cells also acts as antigen-presenting cells for T lymphocytes (64), and they produce pro-inflammatory cytokines that are useful for T-cell hyperactivity and neutrophil priming (**Figure 1**). These mechanisms suggest that B-cell depletion could be a potential target for AAV therapy.

#### Rituximab

Rituximab, an anti-CD20 IgG1 chimeric mouse/human monoclonal antibody, was approved by the U.S. Food and Drug Administration in 2011 for the management of AAV. Rituximab in AAV has been used in two RCTs, the RAVE (Rituximab for the Treatment of Wegener’s Granulomatosis and Microscopic Polyangiitis) trial and the RITUXVAS trial, an international, randomized, open-label trial comparing rituximab-based regimens with standard cyclophosphamide/azathioprine regimens in the treatment of active “generalized” AAV (13, 14). There were several differences between these two trials: the RITUXVAS trial included new patients with severe renal disease, whereas the RAVE trial included new and recurrent patients with well-maintained renal function. Oral cyclophosphamide was used as a comparator in RAVE, and pulsed cyclophosphamide was used in the RITUXVAS trial. In both trials, rituximab was given as four infusions with a body surface area of 375 mg/m^2^, but in the RITUXVAS trial, cyclophosphamide was given in addition to rituximab for two to three cycles. Prednisolone was tapered and discontinued by 5 months in the RAVE trial, but was reduced to 5 mg by 6 months in the RITUXVAS study and continued for the remainder of the study. In the RAVE trial, after 18 months, 39% of patients in the rituximab group and 33% of those in the control group maintained complete remission (65). In that study, there was no significant difference in the total number of serious adverse events between the two groups. In the RITUXVAS trial, the rate of sustained remission was 76% in the rituximab group and 82% in the control group (14). A long-term analysis of these patients showed that relapse occurred at 24 months in 42% of the rituximab group and 36% of the cyclophosphamide group (66). Rituximab can be used in patients who are intolerant to cyclophosphamide, in patients of reproductive age, and in patients who have had substantial prior exposure to cyclophosphamide. However, the efficacy of rituximab monotherapy in severe disease has not been established and there is no consensus on the appropriate dosing regimen.

#### Anti-IL-5 Monoclonal Antibody

One of the hallmarks of EGPA is eosinophilic inflammation. IL-5, a major cytokine that activates eosinophils, has been postulated to be involved in the pathogenesis of EGPA. IL-5 is produced by Th2 cells and induces differentiation and maturation of human eosinophils (67). IL-5 also inhibits eosinophil apoptosis (68).

#### Mepolizumab

Mepolizumab is a humanized anti-IL-5 monoclonal antibody that shows clinical benefit in the management of refractory and relapsing EGPA (15). In patients with EGPA, mepolizumab resulted in a significantly greater number of weeks in remission and a higher proportion of patients in remission compared to a placebo, thus allowing for reduced glucocorticoid use. In 2015, the U.S. Food and Administration expanded the approved use of mepolizumab to treat EGPA. Limitations of the study on which the approval was based were that less than 10% of patients were ANCA positive at baseline and that no analysis of outcomes according to ANCA status was performed.

#### B-Cell Activating Factor Inhibitor (BAFF)

BAFF is also involved in Ig class switching and subsequent antibody production *in vivo*. BAFF promotes B-cell proliferation and splenic B-cell survival *in vitro* (69, 70). Soluble BAFF binds to three different TNF receptors: B cell maturation antigen (BCMA), transmembrane activator, calcium regulator, and cyclophilin ligand interactor (TACI), and BAFF-R (BR3). BCMA and TACI, but not BAFF-R, bind to another B receptor for a proliferation-inducing ligand that is also a cell survival ligand (71). When BAFF binds to its high affinity BAFF-R, the NF-κB pathway (both classical and non-classical pathways) and the MAPK pathway are activated, leading to the expression of genes essential for B cell survival (72).

#### Belimumab

Belimumab is a human monoclonal IgG1 antibody against B-lymphocyte-stimulating factor (B-Lys) against BAFF and is being investigated as a therapeutic option for AAV. B-lymphocyte-stimulating (B-Lys) factor is a cytokine that promotes B-cell survival, maturation, and differentiation, and B-Lys has been observed to be elevated in the serum of patients with AAV, particularly patients with GPA (73, 74). Belimumab has been used as a treatment for Lupus. The BREVAS clinical trial is a phase III multi-center, multinational, randomized and double-blind study evaluating the efficacy and safety of belimumab in combination with azathioprine for the maintenance of remission in GPA and MPA patients (75). This trial demonstrated that the addition of belimumab to a regimen of azathioprine plus low-dose glucocorticoids to maintain remission of AAV did not reduce the risk of recurrent vasculitis. However, patients who received belimumab after remission with rituximab did not experience recurrence of vasculitis. the BREVAS trial has limitations, including sample size (placebo n=52, belimumab n=54). In addition, the number of patients in remission with rituximab who received belimumab was quite low (n=14). Investigation of the maintenance of remission of AAV with belimumab as monotherapy is needed to determine the potential therapeutic benefit of this biologic. Currently, the combination of belimumab and rituximab is being investigated in patients with PR3 ANCA-positive AAV, and the primary endpoint is to compare the time to PR3 ANCA negativity with rituximab alone.

#### Anti-TNF-α Antibody

TNF-α is a multifaceted cytokine that plays a central role in inflammation and leads to the production of a wide range of other pro-inflammatory cytokines and chemokines in the kidney disease (76). In AAV, TNF-α mRNA expression is increased in leukocytes and renal tissue, indicating its involvement in the pathophysiology of the disease (77, 78). These data suggested that TNF-α induces the expression of autoantigens involved in vasculitis on the leukocyte cell membrane, preparing the cells for the effects of ANCAs.

#### Etanercept

Etanercept, a p75 Fc fusion protein against TNF-α, is a biologic agent that has been studied in AAV. A randomized controlled trial (Wegener’s Granulomatosis Etanercept Trial [WGET]) compared the ability of treatment with etanercept 25 mg 2×/week plus standard care with that of placebo plus standard care to maintain clinical remission in patients with GPA (79). Of the 174 evaluable patients, 126 (72.4%) achieved sustained remission, while only 86 (49.4%) remained in remission. There was no significant difference between the etanercept and control groups in the rates of sustained remission (69.7% vs. 75.3%) and sustained low-level disease activity (86.5% vs. 90.6%). Analysis of the relative risk of disease relapse during follow-up also showed that there was no significant difference in the incidence of disease relapse between the etanercept and control groups.

#### Infliximab

Infliximab is a chimeric mouse/human monoclonal antibody against TNF-α and has been used for the treatment of AAV (GPA and MPA). A prospective randomized controlled trial comparing infliximab with rituximab for remission induction in patients with severe refractory GPA showed that both agents were effective in inducing remission, but the general data tended to favor rituximab over infliximab; all non-responders to infliximab were thus switched to rituximab (80). Although no significant results were obtained, the trial’s analyses suggested that (*i*) rituximab has a higher response rate and a higher sustained remission rate, but (*ii*) there are some cases in which infliximab is useful.

#### Adalimumab

Adalimumab is a humanized anti-TNF-α monoclonal antibody that is being studied in a phase II open-label, prospective study in patients with AAV with renal impairment. Adalimumab (40 mg every 2 weeks) plus cyclophosphamide pulsed infusion (compared to cyclophosphamide pulsed infusion alone) showed similar remission-inducing effects and adverse events, but it reduced the dose of glucocorticosteroids in the adalimumab group (81). However, that study had several limitations including a small sample size and the lack of a control group and randomization. At present, all clinical trials of anti-TNF-α antibodies as treatments for AAV have been suspended.

Concerns have been raised regarding the risk of malignancy with anti-TNF-α treatment in AAV, and the results of a subsequent analysis of the WGET trial with an extended period of follow-up (82) suggested that the increased risk of cancer was not significantly different between the intervention and placebo groups compared to the general population, and that this risk could not be attributed solely to etanercept treatment. However, all of the solid malignancies in the etanercept group occurred in patients who received cyclophosphamide, suggesting that etanercept should not be administered after cyclophosphamide because of the increased incidence of malignancy.

#### CTLA4-Ig

CTLA4-Ig, a soluble fusion protein consisting of the extracellular domain of CTLA4 and the CH2-CH3 domain of IgG modified not to bind the Fc receptor, binds to B7.1 and B7.2 with much higher affinity than CD28 and thus serves as an efficient competitive antagonist of the important B7/CD28 When CD28 on the surface of T cells binds to B7.1 (CD80) or B7.2 (CD86) on the surface of activated antigen-presenting cells (APCs) or B cells (83). It activates signaling pathways that promote T cell survival, leading to the formation of a CD40L (CD40+) receptor on T cells. CD40L (CD154) expression is induced in T cells; CD40L interacts with CD40 on APCs, resulting in further upregulation of MHC and B7 and release of cytokines and other inflammatory mediators (84). In addition, CD40L on activated T cells interacts with CD40 on antigen-specific B cells, which induces B cell proliferation and germinal center formation (85, 86). Costimulation-dependent cell-cell interactions within the germinal center lead to B cell maturation through immunoglobulin isotype switching, somatic mutations, clonal expansion of high-affinity B cells, terminal differentiation into plasma cells, and formation of memory B cells that further activate T cells by expressing B7 and acting as APCs (85, 86). which further activate T cells by expressing B7 and acting as APCs (85–87).

#### Abatacept

Abatacept is a fusion protein that fuses the Fc region of IgG1 with the extracellular domain of CTLA4 and inhibits the intracellular co-stimulation of T cells. By containing CTLA4, abatacept blocks the engagement of CD28 with its ligand, thereby inhibiting T-cell activation. Based on the rationale that the blockage of T-cell activation might impact the disease pathogenesis of GPA (88), an open-label trial was conducted to investigate the safety and efficacy of abatacept in patients with non-severe relapsing GPA. The trial’s results demonstrated that abatacept treatment induced remission in the majority of patients (80%) and was well tolerated overall (89). Eleven of the 15 patients (73%) were able to withdraw from prednisone. However, the trial had limitations including the small sample size (n=20) and uncontrolled design. The results cannot be generalized to all GPA patients, and because the study excluded patients with severe disease, no conclusions can be reached about the efficacy of abatacept at the current stage.

### Anti-CD52 Monoclonal Antibody

#### Alemtuzumab

Alemtuzumab is a humanized anti-CD52 monoclonal antibody that selectively reduces the peripheral blood concentrations of T lymphocytes, monocytes, and macrophages. Alemtuzumab has been used to treat AAV patients who have discontinued all immunosuppressive drugs except prednisolone 10 mg/day. A small, uncontrolled study of alemtuzumab administered at doses of 4, 10, and 40 mg on consecutive days was reported, and the patients were followed for an average of 5 years (90). The majority of patients (85%) achieved clinical remission, but a significant proportion of these patients (72%) relapsed at a median period of 9 months after the treatment ended. Adverse events such as severe infections, malignancies, and Grave’s disease have been reported in patients treated with alemtuzumab (90, 91). Alemtuzumab appears to be able to bring refractory and relapsing AAV into remission. Patients treated in the above-described study had a high incidence of adverse events, particularly serious infections, and many received treatment after suffering significant morbidity with a very poor pre-treatment prognosis. Young patients with relapsing or refractory disease before suffering major vital organ damage may benefit from alemtuzumab. Careful monitoring for infections, thyroid disease (in the long term), and malignancies (over the long term) should be performed in all patients treated with alemtuzumab. In order to recommend alemtuzumab as the standard of care for refractory AAV, randomized controlled trials testing the efficacy of alemtuzumab are needed. However, clinical trials of AAV in alemtuzumab have also been suspended.

#### Th17 Inhibitors

Antibodies directed against IL-17 and IL-23 (secukinumab and ustekinumab, respectively) are effectively used in other autoimmune conditions such as psoriasis (92), but no studies of these monoclonal antibodies for the treatment of vasculitis have been conducted despite the existence of a clear rationale.

### C5 and C5a Receptor Blocking Antibodies

#### Eculizumab

Eculizumab, a commercial C5 blocking antibody, binds C5 with picomolar affinity and inhibits its enzymatic activation by C5 convertases, possibly through steric hindrance (93). Antibodies directed against C5 have shown remarkable clinical benefits for the diseases paroxysmal nocturnal hemoglobinuria (94) and atypical hemolytic uremic syndrome (95), but a study in AAV was withdrawn without a detailed description (no reference).

#### Avacopan

Avacopan (formerly known as CCX168) is a novel, orally available, highly selective human C5aR antagonist with no other known pharmacological effects (93). Avacopan does not inhibit the interaction of C5a with its associated receptor C5L2 (also called C5aR2), and avacopan is thought to have anti-inflammatory properties (96). Abacopan has also been shown to exert a protective effect in a mouse model of anti-MPO-induced glomerulonephritis (97). Abacopan, a specific C5aR antagonist, does not inhibit the formation of the terminal complement complex or the membrane attack complex C5b-9, which are required for the elimination of pathogenic endophytic bacteria such as *Neisseria meningitidis*.

The CLEAR and CLASSIC Phase II trials confirmed that treatment with avacopan 30 mg 2×/day is safe and can be used in place of glucocorticoids for the induction of AAV remission (98, 99). The results of the ADVOCATE trial, a large randomized trial comparing avacopan+placebo with standard glucocorticoid therapy, both in combination with cyclophosphamide or rituximab, are awaited for approval and will probably lead to a major breakthrough in the treatment of AAV (100).

## Conclusion

Biologics play an important role in inducing and maintaining clinical remission in severe ANCA-associated vasculitis. The improved understanding of the disease process gained over the past decade has led to the identification of multiple new targets and strategies to treat this deadly disease. The development of tools to assess the pathogenesis of vasculitis and extensive experience with a series of clinical trials has established a foundation from which new agents can be evaluated.

Rituximab is indicated for the induction of remission and the management of severe recurrent GPA/MPA, although the role of preemptive, fixed-interval therapy with rituximab in maintaining remission has been suggested. Mepolizumab has shown efficacy in the management of severe refractory and relapsing EGPA. Belimumab and avacopan are undergoing clinical trials testing their efficacy and safety in AAV. Although anti-TNF-α biologics are not currently recommended as remission-inducing therapy for AAV, case reports and nonrandomized, open-label trials have provided data that justify the use of these biologics in certain relapsed and refractory cases. However, concerns remain regarding the incidence of malignancies. Abatacept has been well tolerated and a high percentage of patients have achieved disease remission and have been able to discontinue prednisone. Nevertheless, further studies are required to establish the safety and efficacy of these biologics. The clinical efficacy of alemtuzumab has been suggested, but a significant proportion of relapses and a high rate of adverse events such as severe infections were reported for this biologic. All clinical trials for AAV have been suspended at this time.

Strategies to reduce the toxicity associated with treatment without compromising efficacy are important goals for future research. Treatments need to be optimized and customized according to the severity of the patient’s disease and the risk of recurrence. This will require a better understanding of the etiology of AAV and the development of powerful biomarkers.

In conclusion, the potential benefits, adverse effects, and risks of biologic agents in the management of AAV should be considered prior to the administration of these drugs for the induction and maintenance of disease remission.

## Author Contributions

The author confirms being the sole contributor of this work and has approved it for publication.

## Conflict of Interest

The author declares that the research was conducted in the absence of any commercial or financial relationships that could be construed as a potential conflict of interest.
